# Differences between bone age and chronological age in patients with open physes and anterior cruciate ligament injury using a Magnetic Resonance Imaging bone age assessment tool of the knee

**DOI:** 10.1002/jeo2.70316

**Published:** 2025-07-02

**Authors:** Alberto Grassi, Claudio Rossi, Emanuele Altovino, Luca Ambrosini, Federico Maria Adravanti, Amir Assaf, Kyle Borque, Stefano Zaffagnini

**Affiliations:** ^1^ II Clinica Ortopedica e Traumatologica, IRCCS Istituto Ortopedico Rizzoli Bologna Italy; ^2^ Dipartimento di Scienze Biomediche e Neuromotorie (DIBINEM) University of Bologna Bologna Italy; ^3^ Department of Orthopaedic Surgery Houston Methodist Hospital Houston Texas USA

**Keywords:** ACL, physeal sparing, skeletal age, skeletally immature

## Abstract

**Purpose:**

This study examines the efficacy of the Pennock Bone Age Atlas, which utilizes knee magnetic resonance imaging (MRI), in accurately assessing skeletal maturity in paediatric patients with anterior cruciate ligament (ACL) injuries. Specifically, it investigated the differences between chronological and bone ages in skeletally immature patients to inform clinical decision‐making for ACL reconstruction.

**Methods:**

A total of 79 skeletally immature patients with ACL injuries, treated between February 2022 and June 2024, were included in this study. Bone age was assessed using the Pennock Atlas. The differences between chronological and bone ages were calculated, and the remaining growth potential was estimated. Statistical analyses were conducted to identify any significant discrepancies in growth expectations based on these measures.

**Results:**

On average, bone age closely matched chronological age. However, 25.3% of patients showed discrepancies greater than 1 year, particularly in the 12–14 age group. About 15% of patients who were expected to have no further growth according to chronological age had remaining growth potential when assessed by bone age, while 11% showed completed growth despite their chronological age suggesting otherwise. This discrepancy emphasizes the need to consider skeletal maturity rather than relying solely on chronological age in clinical decisions.

**Conclusion:**

In the setting of ACL injury in skeletally immature patients, the Pennock Bone Atlas was able to accurately detect differences between bone age and chronological age, identifying a mismatch of more than 1 year between the two ages in up to 25% of cases. This information can be used to guide surgeons in choosing different treatments optimized for the patient's growth potential.

**Level of Evidence:**

Level IV case series.

**Abbreviations**
ACL: anterior cruciate ligament
MRI: magnetic resonance imaging

## INTRODUCTION

As younger generations engage in more strenuous sports, a noticeable rise in the incidence of anterior cruciate ligament (ACL) injuries among paediatric populations has been reported [9, 11, 16].

Surgical treatment is indicated in case of clinical instability to prevent subsequent meniscal injury and degenerative changes [8, 10].

Since young individuals are still developing, physeal‐sparing ACL reconstruction techniques have been developed to avoid growth plate damage and subsequent growth disturbances [3, 4]. Decisions regarding the need for physeal‐sparing ACL reconstruction techniques are commonly based on the skeletal age of these patients, as chronological age is not a reliable reference. In fact, while the majority of adolescent patients are still developing and present with open physes, many have already reached maturity. Conversely, some may not reach maturity despite appearing mature for their chronological age. To properly assess skeletal age in this population, various methods and atlases have been developed [7, 12, 13, 14]. Regarding bone age assessment, the most widely utilized reference is the atlas of Greulich and Pyle, which is based on a single left‐hand‐wrist radiograph [7].

Despite its widespread use, it has the limitation of not considering that skeletal age assessed from wrist radiographs could vary from that assessed with knee radiographs and thus this approach could not be appropriate when treating knee pathologies [1]. Moreover, this method requires the execution and interpretation of an additional radiological imaging not directly related to the involved joint. To overcome these limitations, Pennock et al. [13] developed an atlas of knee magnetic resonance imagings (MRIs) spanning the paediatric and adolescent years that enabled accurate assessment of skeletal age and that had a similar reliability to the Greulich and Pyle atlas. A further study [14] created a shorthand approach to determine skeletal maturity based on the previous atlas, with an excellent interrater and intra‐rater reliability, even superior to the hand atlas. This shorthand atlas was based on the interpretation of several MRI features, such as the ossification status of the epiphysis and tibial tubercle, the subchondral ossification and the femoral and tibial growth cartilage closure.

However, neither the Pennock atlas nor its shorthand has been created and validated in a general healthy population, and its application in a clinical setting of ACL‐injured patients is lacking.

Thus, the aim of the present study was to apply the Bone Age Atlas based on knee MRI to the clinical scenario of ACL injury in skeletally immature patients and investigate the differences between chronological and bone ages based on this novel method. The hypothesis was that there is a relevant number of skeletally immature patients with ACL injury who exhibit a difference of 1 year or more between bone and chronological ages.

## METHODS

All skeletally immature patients with ACL rupture treated at the Rizzoli Orthopaedic Institute Hospital between February 2022 and June 2024 by a single surgeon (G.A.) were prospectively enroled in this study. Diagnosis of ACL rupture was based on medical history, clinical examination and MRI assessment. Patients were considered ‘skeletally Immature’ if either the tibial or femoral physis was open on their knee MRI. No exclusion criteria were applied. Patients were enroled whether they were treated surgically or non‐operatively.

### Bone age MRI evaluation

The MRIs of all patients were assessed by a single surgeon who specializes in adult and paediatric ACL reconstruction (G.A.). The shorthand Pennock Atlas based on knee MRI was used to determine bone age [13, 14]. According to the aforementioned Atlas, several MRI features were assessed, each of which corresponded to a specific bone age for either males or females (Table 1).

**Table 1:** Characteristics of the knee MRI used to determine bone age according to the Shorthand of Pennock Atlas [14].

Bone age according to the different features of the Pennock Atlas Shorthand		
	Bone age	
Features	Females	Males
Tibial tubercle extension + no apophyseal ossification	9 years	11 years
Tubercle ossification + no tubercle ‘crack’	10 years	12 years
Tubercle ‘crack’ + tubercle ‘not fully ossified’	10.5 years	13 years
Tubercle fully ossified + ‘oreo’ sign present	11.5 years	14 years
‘Oreo’ sign disappeared + physis still visible	13 years	15 years
Femoral physis completely visible + partial closure of tibial physis	14 years	16 years
Partial closure of femoral physis + partial\complete closure of tibial physis	15 years	17 years

Abbreviation: MRI, magnetic resonance imaging.

#### Tibial tubercle extension of the epiphysis

Assessed on the sagittal images, it appears when the ossification of the proximal tibial epiphysis is extending downward toward the tibial tubercle; however, the ossification centre of the tibial tuberosity itself is not present yet.

#### Presence of tibial tubercle apophyseal centre

Assessed on the sagittal images, it is identified when a discrete ossification nucleus inferiorly respect to the tibial physis is present. It indicates that the tibial tubercle is starting to develop, and that the maturation of the proximal epiphysis is almost complete.

#### Presence of tibial tubercle ‘crack’

Assessed on the sagittal images, it is identified as a thin hypointense line separating the tibial epiphysis and the tibial tubercle apophysis. It indicates that the two structures are close to complete fusion with each other.

#### ‘Oreo’ sign

Assessed on the sagittal images at the level of the femoral condyles, it is defined as a laminated appearance of the subchondral epiphyseal cartilage, usually as black‐grey‐black layers. When the subchondral cartilage presents as a single black layer, the ‘Oreo’ sign is considered to have disappeared.

#### Tibial and femoral growth cartilage status

Evaluated on coronal images, the course of growth cartilage is assessed from the peripheral portion to the centre of the bone. According to its status, the cartilage could be labelled as ‘completely visible’, ‘partially closed’ (usually in its central portion) and ‘completely closed’.

Since MRIs were performed in different centres and with different protocols, it was not possible to standardize the assessment. However, the latter features used to determine the bone age did not require a specific protocol and the available slices, with a thickness between 2 and 4 mm, were used. Thus, each of these factors was evaluated and then the shorthand Pennock Atlas was utilized to determine the patient's ‘bone age’.

The chronological age at time of MRI was subtracted from the bone age at time of MRI, in order to identify the difference between the two. Positive values identified patients whom ‘bone age’ was older than their chronological age, while negative values were for patients whose bone age were younger than their chronological age.

Two values were calculated for years of remaining growth, one by subtracting the chronological age and the other by subtracting the bone age from 16 years in males, and from 14 years in females. Negative values were considered as zero, as this meant that no further growth was expected.

### Statistical analysis

Statistical analysis was performed with MedCalc (MedCalc Software). A power analysis was performed to identify the number of samples required to detect a significant difference between bone and chronological ages of 0.5 ± 0.5 years with an alpha error of 0.05 and a beta‐power of 0.05. A total of 54 patients were required according to this calculation.

Continuous variables were reported as mean ± standard deviation, while categorical variables were reported as absolute number and proportion of the total sample. Independent sample *t*‐test was used to compare the continuous variables, and Fisher's exact test was used to compare dichotomous categorical variables. Statistical significance was set at *p* < 0.05.

## RESULTS

### Patient characteristics

A total of 79 skeletally immature patients (83% males and 17% females) were diagnosed with an ACL injury and were included in this study (Table 2).

**Table 2:** Demographic details of the included patients.

Demographic details	
Sex (M/F)	66 (83%)/13 (17%)
Chronological age	13.9 ± 2.2 years
Males	13.9 ± 2.3 years
Females	13.8 ± 1.5 years
Skeletal age	14.0 ± 2.3 years
Males	14.0 ± 2.5 years
Females	14.3 ± 1.2 years
Difference between bone and chronological age	0.1 ± 0.9 years
Years of remaining growth based on chronological age	1.9 ± 2.3 years
Years of remaining growth based on bone age	1.9 ± 2.2 years

**Figure 1:**
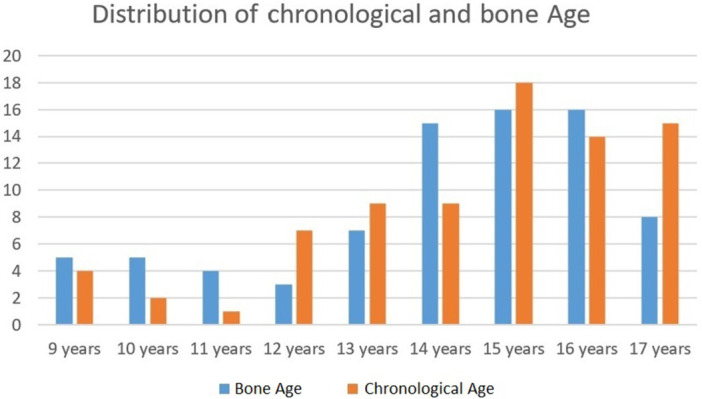
Distribution of bone age (blue bars) and chronological age (orange bars) according to ages.

The chronological and bone ages at time of MRI were 13.9 ± 2.2 years and 14.0 ± 2.3 years, respectively, with a non‐significant difference (*p* > 0.05) of 0.1 ± 0.9 years (range −2.2 to +2.5 years). No significant differences were present in either bone or chronological age between males and females (*p* > 0.05). The most represented chronological and bone age was 15 years (Figure 1), characterized by open tibial and femoral physis, fully ossified anterior tibial apophysis and disappeared ‘oreo sign’.

### Differences between chronological and bone ages

**Figure 2:**
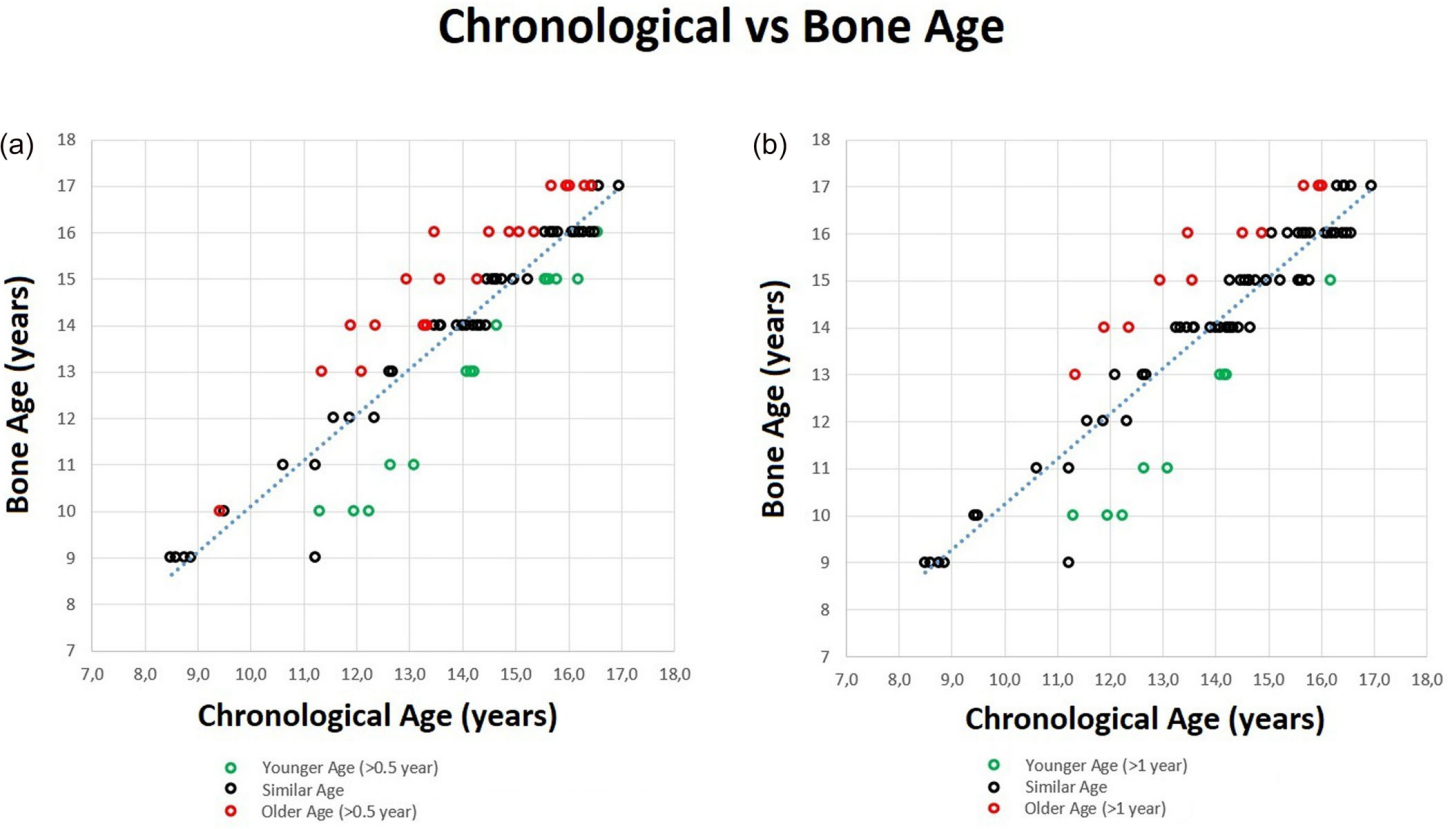
Scatter plot graphic with bone age on the *Y*‐axis and chronological age on the *X*‐axis. Black dots represent similar bone and chronological age, green dots younger bone age and red dots older bone age, with 0.5 years cut‐off (Graph a) and 1.0 year cut‐off (Graph b).

A total of 20 out of 79 patients (25.3%) had a difference between chronological and bone ages superior to ±1 year. With respect to the chronological age, 11 out of 79 patients (13.9%) had older bone age and 9 out of 79 patients (11.4%) had younger bone age (Figure 2, Table 3). A total of 3 out of 14 patients (21%) with <12 years, 8 out of 18 patients (44%) between 12 and 14 years and 9 out of 47 patients (19%) with >14 years had a difference superior to 1.0 year between chronological and bone ages.

**Table 3:** Differences between bone age and chronological age.

	Patients	%
Age difference <0.5 year	44/79	55.7
Age difference ≥0.5 year	35/79	44.3
Older bone age	20/79	25.3
Younger bone age	15/79	19.0
Age difference <1.0 year	59/79	74.7
Age difference ≥1.0 year	20/79	25.3
Older bone age	11/79	13.9
Younger bone age	9/79	11.4

Differently, 35 out of 79 patients (44.3%) had a difference between chronological and bone ages superior to ±0.5 years. With respect to chronological age, 20 out of 79 patients (25.3%) had older bone age and 15 out of 79 patients (19.0%) had younger bone age (Figure 2, Table 3). A total of 3 out of 14 patients (21%) with <12 years, 11 out of 18 patients (61%) between 12 and 14 years and 20 out of 47 patients (43%) >14 years had a difference superior to 0.5 years between chronological and bone ages.

Considering only patients with chronological and bone ages mismatch, the average difference between bone age and chronological age was −1.5 ± 0.4 years for patients who had bone age younger than chronological age and +1.6 ± 0.5 years for patients who had bone age older than chronological age.

### Analysis of remaining knee growth

The average remaining growth at the time of ACL injury was 1.9 ± 2.3 years (range: 0–7.5 years) and 1.9 ± 2.2 years (range: 0–7.0 years) according to chronological age and bone age, respectively, with no significant differences between the two values (*p* < 0.05). The average remaining growth for the subgroup of patients with at least 1 year of remaining growth was 3.1 ± 2.1 years.

According to chronological age, 33 patients (41.7%) had no remaining skeletal growth; however, 5 of them (15.2%) had at least 1 year of remaining skeletal growth according to bone age assessment. Differently, among the 27 patients who had 1–3 years of growth according to chronological age, 3 patients (11.1%) had no remaining growth according to bone age.

## DISCUSSION

The study represents the first clinical application of a Bone Age Atlas based on knee MRI, on a specific population of skeletally immature patients with ACL injury. The findings support the hypothesis that this knee MRI Bone Age Atlas is able to identify significant differences between chronological and skeletal age, and that a relevant percentage of skeletally immature patients with ACL injury have a relevant mismatch between the two ages, with possible implications in the surgical decision‐making process. In fact, it is crucial to accurately determine skeletal maturity in paediatric patients with ACL tears, as these patients will undergo ligament reconstruction surgery, which represents a risk of growth plate damage from the creation of bone tunnels, potentially resulting in future limb alignment and length defects [4, 5].

Historically, tools like the Tanner scale have been used to estimate physiological age, however, such methods have shown poor reliability compared to bone age assessments [2] and orthopaedic surgeons often find it difficult to assess Tanner stages accurately [15] The Greulich and Pyle atlas, based on hand‐wrist radiographs, is widely used but may not reflect skeletal development in other joints, such as the knee [1] and requires an additional radiographs with radiation exposure, when knee pathologies are treated. These limitations were addressed by the Pennock et al. atlas [13], which showed a reliability as reliable as the Greulich and Pyle method in assessing skeletal age. Politzer et al. [14] later simplified this with a shorthand MRI approach, focusing on key markers of skeletal maturity, such as tibial tubercle ossification and growth plate closure, offering high interrater and interrater reliability.

**Figure 3:**
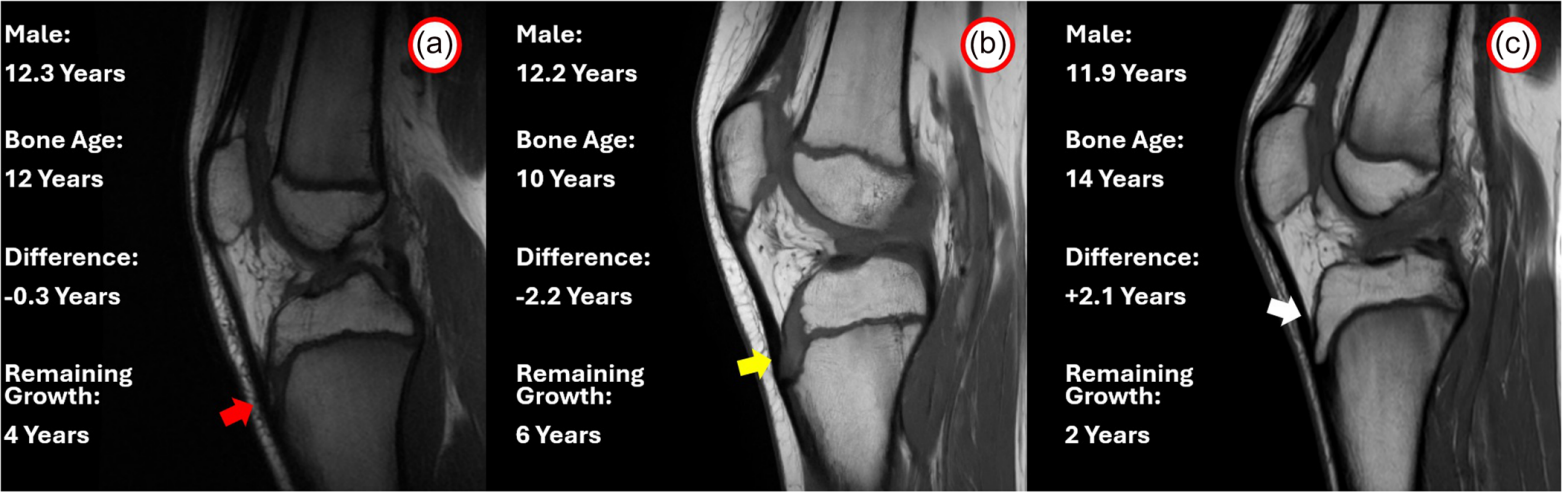
Example of mismatch between chronological and bone ages in three patients with the same chronological age (12 years). Male patient with similar bone and chronological ages (12 years), as demonstrated by the presence of tibial tubercle apophyseal centre (red arrow) (a). Male patients with younger bone age (10 years), as demonstrated by the absence of tibial tubercle extension to the epiphysis (yellow arrow) (b). Male patients with older bone age (14 years) as demonstrated by the complete closure of tibial tubercle ossification (white arrow) (c). These examples show a possible difference of 4 years of remaining growth in patients with the same chronological age.

**Figure 4:**
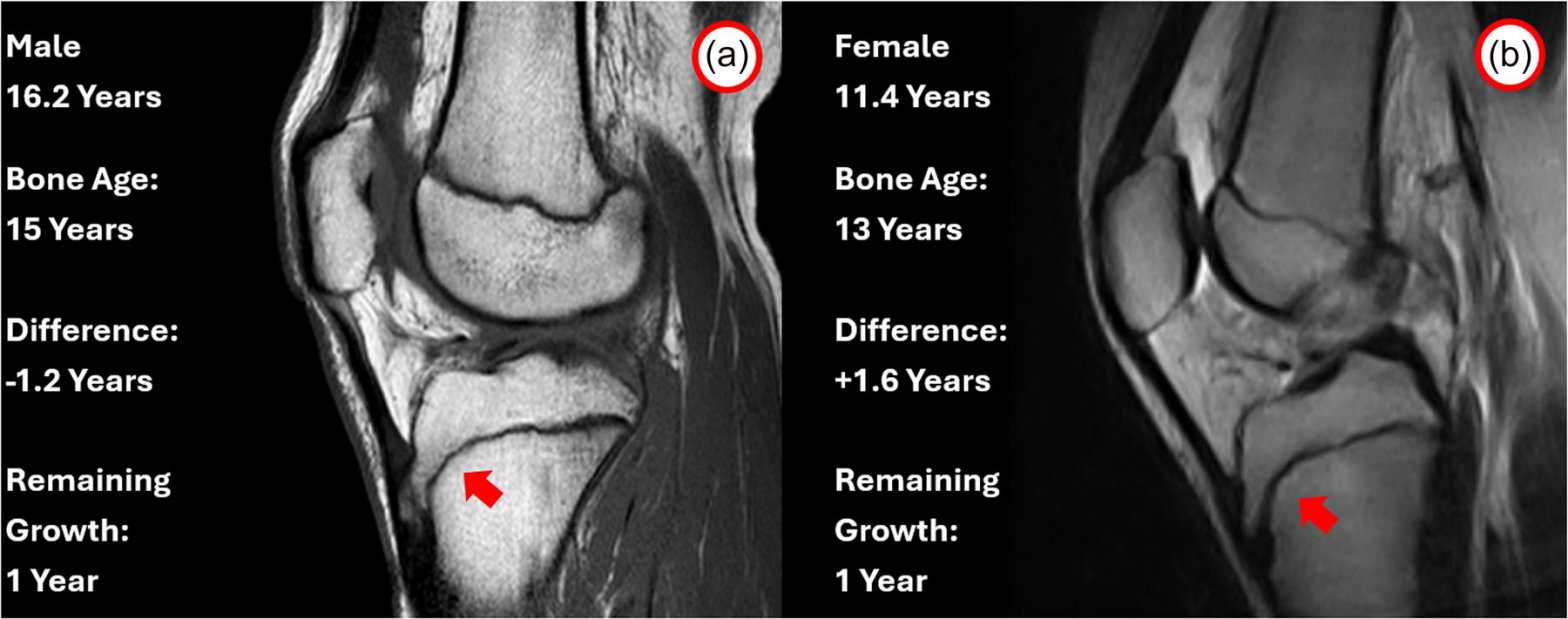
Example of two patients with similar remaining growth according to bone age assessment (1 year) but different chronological age. In this case, a 16.2‐year‐old male (a) and an 11.4‐year‐old female have the same ossification status characterized by complete closure of tibial tubercle ossification (red arrow) and open femoral and tibial growth plates. Despite the difference of nearly 5 years in chronological age, the remaining growth is similar.

The results of this study show that skeletal age closely matches chronological age in most patients, with no significant difference between males and females. However, 25.3% of the patients showed a difference greater than 1 year. This discrepancy is mostly represented in patients between the ages of 12 and 14 years; in fact, 44% of the patients in this age group demonstrated a difference between bone age and chronological age greater than one year. These data suggest that patients in this age group have highly variable bone ages, and thus, it is crucial to identify bone age as accurately as possible to avoid treatments that could impact their remaining growth. It is common that patients with the same chronological age could have a wide range of remaining growth (Figure 3) or that patients several years apart could exhibit the same remaining growth (Figure 4).

In this context, the article highlights an interesting finding: approximately 15% of patients who, based on their chronological age, were expected to have completed their growth, still had at least 1 year of remaining growth when evaluated by bone age. This highlights the importance of assessing skeletal age rather than depending exclusively on chronological age, particularly when the surgical treatment could impact future growth. In these cases, despite chronological age, a physeal‐sparing technique could be considered and adapted to the bone maturity and remaining growth [6].

**Figure 5:**
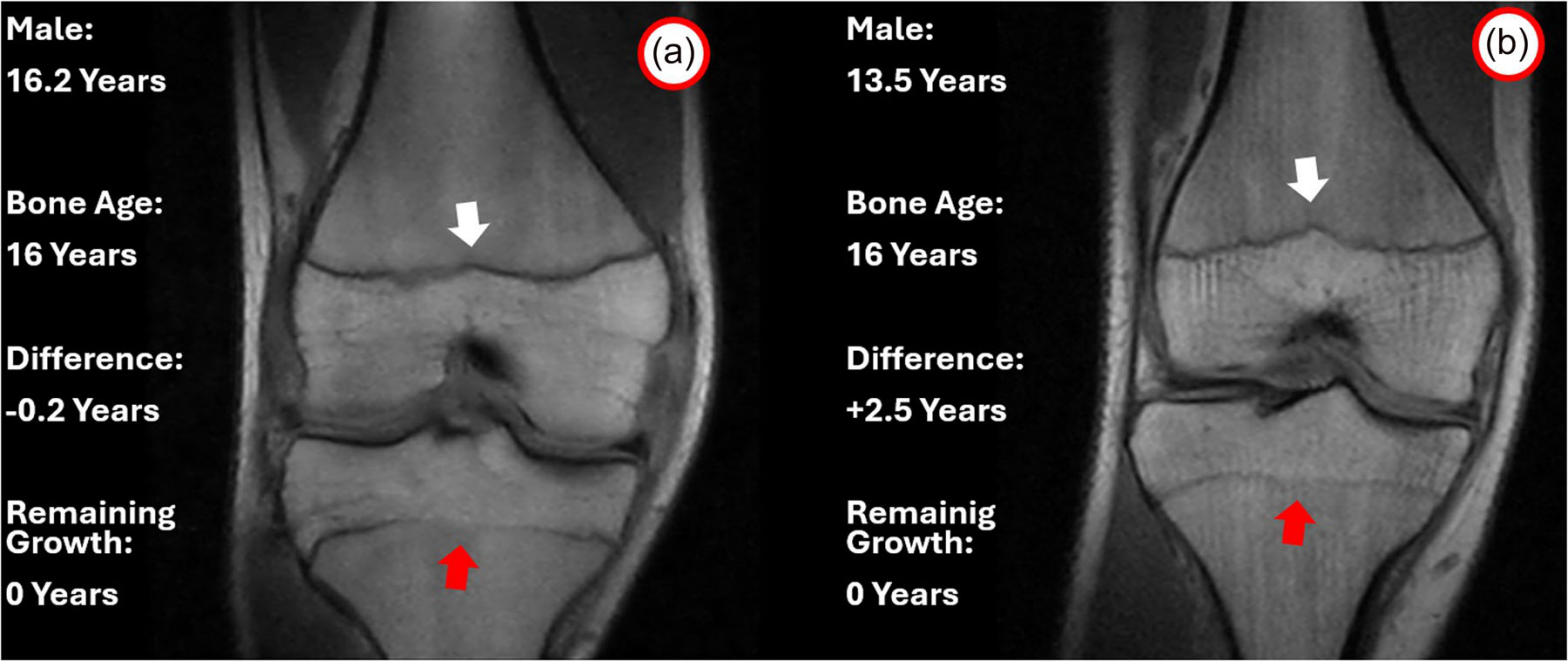
Example of two patients with no remaining growth according to bone age assessment. The 16.2‐year‐old patient has similar bone and chronological ages, with 0 years of remaining growth, as demonstrated by open femoral physis (white arrow) and closing tibial physis (red arrow) (a). The same ossification status and no remaining growth was present in a 13.5‐year‐old male, where at least 2 years of remaining growth were expected according to chronological age (b).

Conversely, a significant 11% of patients who, based on their chronological age, were expected to grow for another 1–3 years, had actually completed their growth and could be treated as adults (Figure 5).

This is an important and clinically useful finding, as it highlights that in ACL reconstruction, there is often concern about treating patients with a young chronological age, who, however, may have already reached their skeletal maturity. These results allow surgeons to consider different operative treatments even in patients with the same chronological age. Patients with a discrepancy where bone age indicates ongoing growth should receive treatments that minimize long‐term impact on joint development. Conversely, patients whose chronological age suggests remaining growth but whose bone age indicates a mature bone age can undergo standard procedures. The study highlights how the Pennock Shorthand MRI Atlas can effectively identify differences between bone and chronological ages, providing a precise evaluation of skeletal maturity.

Despite the promising findings, this study has several limitations. First, the number of patients involved in the study is limited. This investigation should be conducted on a larger and more representative sample to obtain more standardized results. Moreover, there is a significant gender imbalance (83% males vs. 17% females), which could affect the validity of the results for both groups.

Second, while the Pennock Atlas was designed for knee pathology in general, the study is focused only on a specific group of patients affected by ACL injury. Therefore, further studies are needed to confirm that the Atlas can identify discrepancies between skeletal and chronological age with the same accuracy in different knee conditions.

Finally, this study evaluates the use of Pennock Bone Atlas to detect the difference between bone and chronological age in patients with ACL injury without investigating, however, its usefulness in clinical practice. There is no demonstration of how treatments could vary when using this Atlas in clinical practice or how it could improve long‐term outcomes for patients with ACL injuries. Therefore, more studies are needed to see how using the Pennock Atlas could affect treatment decisions for patients.

In conclusion, the Pennock Bone Age Atlas has been demonstrated to be a valid method to determine the difference between skeletal age and chronological age in patients with ACL injury, giving valuable information on the remaining growth of immature patients. Further studies are needed to apply this method to a larger cohort of patients and to evaluate how the clinical use of this Atlas can influence surgical treatment decisions and long‐term outcomes of patients undergoing ACL reconstruction surgery.

## CONCLUSION

In the setting of ACL injury in skeletally immature patients, the Pennock Bone Atlas was able to accurately detect differences between bone age and chronological age, identifying a mismatch of more than 1 year between the two ages in up to 25% of cases. This information can be used to guide surgeons in choosing different treatments optimized for the patient's growth potential.

## AUTHOR CONTRIBUTIONS

Alberto Grassi, Stefano Zaffagnini and Kyle Borque had the initial idea of the study and supervised the work by reviewing the paper and conducting the decision‐making processes. Emanuele Altovino, Luca Ambrosini and Assaf Amir contributed to the writing of the article and the statistical analysis. Claudio Rossi and Federico Maria Adravanti contributed to the article by making the MRI analysis and writing the introduction and abstract.

## CONFLICT OF INTEREST STATEMENT

Alberto Grassi: Smith & Nephew: Not paid consultant. Stefano Zaffagnini: DePuy, A Johnson & Johnson Company: Paid presenter or speaker, paid consultant; European Society of Sports Traumatology Knee Surgery and Arthroscopy (ESSKA): Board or committee member; International Society of Arthroscopy, Knee Surgery, and Orthopaedic Sports Medicine (ISAKOS): Board or committee member; Journal of Experimental Orthopaedics (JEO): Editorial or governing board; Smith & Nephew: Paid presenter or speaker, paid consultant. Kyle Borque: CONMED Linvatec: Research support; Mitek: Paid consultant; Xiros Inc: Paid consultant; Research support. The remaining authors declare no conflicts of interest.

## ETHICS STATEMENT

Not applicable due to the observational nature of the study on already available clinical and radiological data.

## Data Availability

The data that support the findings of this study are available on request from the corresponding author. The data are not publicly available due to privacy restrictions.
